# Effect of dental metal artifact conversion volume on dose distribution in head‐and‐neck volumetric‐modulated arc therapy

**DOI:** 10.1002/acm2.13101

**Published:** 2020-11-23

**Authors:** Kenta Kitagawa, Hitoshi Ikushima, Motoharu Sasaki, Shunsuke Furutani, Takashi Kawanaka, Akiko Kubo, Chisato Tonoiso, Takaharu Kudoh, Yosuke Kano, Akira Tsuzuki

**Affiliations:** ^1^ Graduate School of Health Sciences Tokushima University Tokushima 770‐8503 Japan; ^2^ Department of Therapeutic Radiology Institute of Biomedical Sciences Tokushima University Graduate School Tokushima 770‐8503 Japan; ^3^ Department of Radiology and Radiation Oncology Institute of Biomedical Sciences Tokushima University Graduate School Tokushima 770‐8503 Japan; ^4^ Department of Oral and Maxillofacial Surgery Institute of Biomedical Sciences Tokushima University Graduate School Tokushima 770‐8503 Japan; ^5^ Department of Radiological Technology Kochi Medical School Hospital Nankoku‐shi Kochi 783‐8505 Japan

**Keywords:** dental metal artifact, head‐and‐neck, volumetric‐modulated arc therapy

## Abstract

**Purpose:**

During treatment planning for head‐and‐neck volumetric‐modulated arc therapy (VMAT), manual contouring of the metal artifact area of artificial teeth is done, and the area is replaced with water computed tomography (CT) values for dose calculation. This contouring of the metal artifact areas, which is performed manually, is subject to human variability. The purpose of this study is to evaluate and analyze the effect of inter‐observer variation on dose distribution.

**Methods:**

The subjects were 25 cases of cancer of the oropharynx for which VMAT was performed. Six radiation oncologists (ROs) performed metal artifact contouring for all of the cases. Gross tumor volume, clinical target volume, planning target volume (PTV), and oral cavity were evaluated. The contouring of the six ROs was divided into two groups, small and large groups. A reference RO was determined for each group and the dose distribution was compared with those of the other radiation oncologists by gamma analysis (GA).

As an additional experiment, we changed the contouring of each dental metal artifact area, creating enlarged contours (L), reduced contours (S), and undrawn contours (N) based on the contouring by the six ROs and compared these structure sets.

**Results:**

The evaluation of inter‐observer variation showed no significant difference between the large and small groups, and the GA pass rate was 100%. Similar results were obtained comparing structure sets L and S, but in the comparison of structure sets L and N, there were cases with pass rates below 70%.

**Conclusions:**

The results show that the artificial variability of manual artificial tooth metal artifact contouring has little effect on the dose distribution of VMAT. However, it should be noted that the dose distribution may change depending on the contouring method in cases where the overlap between PTV and metal artifact areas is large.

## 1 Introduction

Intensity‐modulated radiation therapy (IMRT) is an irradiation method in which the dose distribution can be customized for a complicated target shape by adjusting the dose in the irradiation field.^1^ A site adjacent to the tumor and normal tissue is a good indication for IMRT.^2^ Head‐and‐neck cases are one of the sites suitable for IMRT.^3^ In head‐and‐neck cases, there is an organ at risk (OAR), such as the spinal cord, brainstem, eyeball, optic nerve, or parotid gland, near the primary tumor. In recent years, volumetric‐modulated arc therapy (VMAT) has been used clinically as an advanced type of IMRT. In VMAT, the radiation intensity is modulated by dynamically changing the aperture shape of the multi‐leaf collimator (MLC) and the radiation output per gantry rotation angle.^4^ The dose distribution obtained by VMAT is similar to that of IMRT.^5^, ^6^ Significantly improved treatment efficiency has been reported for VMAT compared to IMRT.^7^

Dental metal artifacts are often seen in patients with head‐and‐neck cancer when performing treatment planning simulator computed tomography (CT). Artifacts caused by the presence of dentals affect the calculation accuracy when attempting to obtain an accurate dose distribution.^7^, ^8^ In recent years, several techniques, such as metal artifact reduction (MAR), have been reported^9^, ^10^, ^11^, ^12^ to reduce the influence of metal artifacts during CT imaging. However, the MAR function is not available in all facilities that perform radiotherapy. Fixed multi‐gantry IMRT does not set the gantry angle orientation if there is a metal artifact in front of the main lesion on the x‐ray entrance side. However, to avoid having the beam incident in the direction of the dental metal artifact when performing VMAT requires increasing the number of arcs to design the same dose distribution as would otherwise be delivered. The method of increasing the number of arcs eliminates the time reduction advantage of VMAT. Therefore, many facilities that perform VMAT do not limit the beam. In general, during VMAT treatment planning, the treatment planner manually performs contouring on the dental metal artifact region and replaces the region with the CT value of water to calculate the dose. Artificial variability may occur in the manual contouring of dental metal artifacts. However, there are no previous studies to date on the effects of artificial variability in dental metal artifact contouring on dose distribution. In addition, because there are no reports on the criteria for contouring metal artifacts, the contouring method may differ greatly depending on the treatment planner. Therefore, the purpose of this study is to clarify the effect of artificial variability of manual dental metal artifact contouring on the dose distribution.

## 2 Methods

### 2.A Patients

Thirty cases of oropharyngeal cancer who underwent VMAT at our institution between November 2014 and March 2019 were included. However, we excluded five cases in which no dental metal artifacts occurred. The reason for targeting oropharyngeal cancer was that the sites of dental metal artifacts were closer to the gross tumor volume (GTV), clinical target volume (CTV), and planning target volume (PTV) than in other head‐and‐neck cases. This study was approved by the Medical Research Ethics Review Committee of our institution.

### 2.B Equipment and treatment planning

Treatment planning CT images were acquired using an Optima CT580W (General Electric Medical Systems, Waukesha, WI, USA). True Beam (Varian Medical Systems, Palo Alto, CA, USA) was used as the medical linear accelerator, and the energy was 6 MV x ray. The treatment planning system (TPS) used was Eclipse (Varian Medical Systems, Palo Alto, CA, USA) version 11.0.31. The VMAT was set to 2‐arc, 181º–179º clockwise, and 179º–181º counterclockwise. The collimator angles were 350º and 90º, and the maximum dose rate was 600 MU/min. The prescription dose was 70 Gy/35 fractions, and the dose was 95% of the PTV70 volume (D95%). The dose calculation algorithm used was the anisotropic analytical algorithm (AAA). To evaluate the dose distribution, 3DVH (Sun Nuclear Corporation, Melbourne, FL, USA), which is software attached to the three‐dimensional dose verification system ArcCHECK (Sun Nuclear Corporation, Melbourne, FL, USA), was used.

### 2.C Difference in dose distribution due to inter‐observer error

#### 2.C.1 Contouring of dental metal artifact area

**Fig 1:**
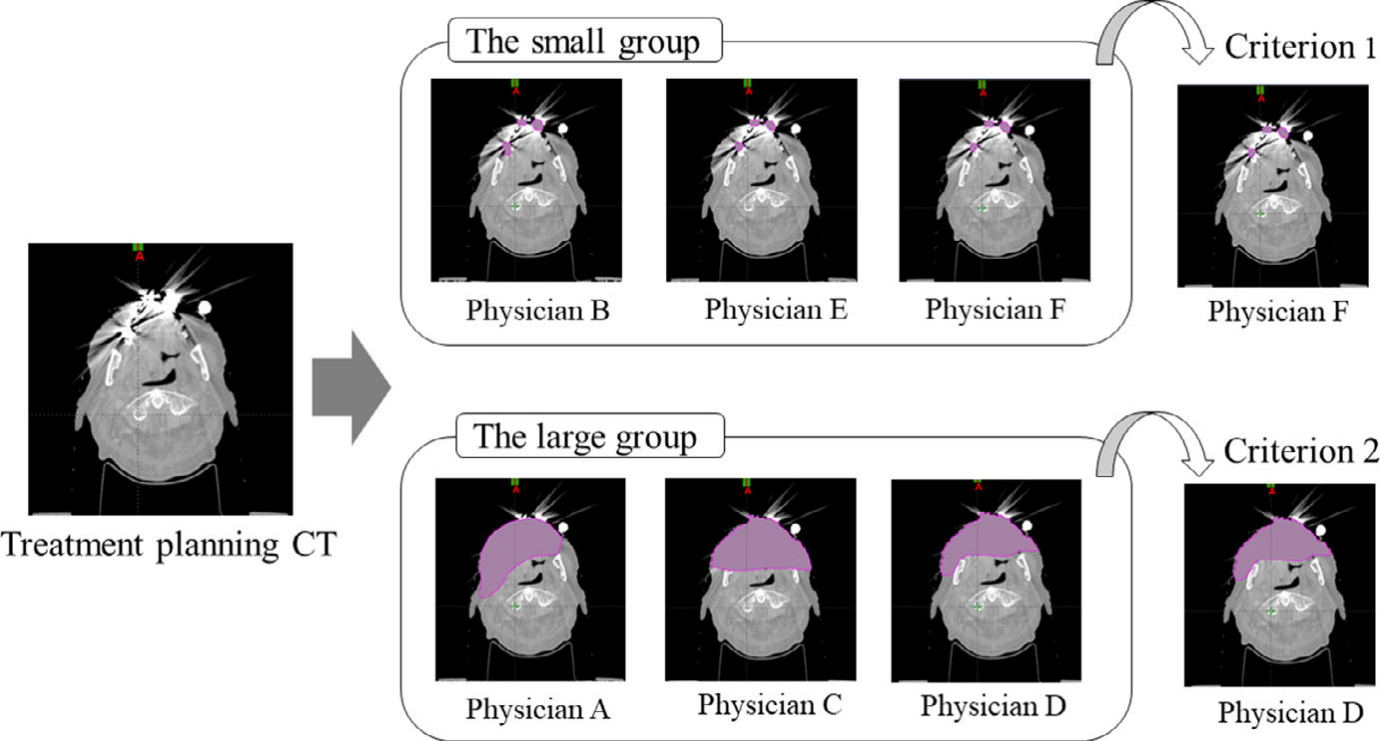
Selection of Criterion 1 and Criterion 2.

Based on the original treatment plan CT image for 25 cases of cancer of the oropharynx, six radiation oncologists (denoted Physician A, Physician B, Physician C, Physician D, Physician E, and Physician F) performed the dental metal artifact area contouring. The structure name of the dental metal artifact area that the radiation oncologist identified by contouring is metal. The metal was replaced with the CT value of water, and other structures remained unchanged and were used in the actual clinical radiotherapy. Therefore, six structure sets were created by the six radiation oncologists. In this study, we defined two groups. The small group was defined as Physicians B, E, and F, who contoured only the areas where dentures were present (Fig. 1). The large group was defined as Physicians A, C, and D, who contoured the streaky artifact area and the dark band area (Fig. 1).

#### 2.C.2 Recalculation of the dose distribution

The dose distribution was transferred from the original treatment plan to the six structure sets created by the six radiation oncologists and recalculation was performed. The original treatment plan was not changed in this study, and no retreatment plan was performed. The original treatment plan and the isocenter of the six structure sets were the same; only the dose distribution was transposed.

#### 2.C.3 Evaluation method

Criterion 1 was defined as the contouring of Physician F, whose volume of dental metal artifact contouring was closest to the mean among the three radiation oncologists in the small group. Similarly, Criterion 2 was set as the contouring of Physician D, whose volume of dental metal artifact contouring was closest to the mean value among the three radiation oncologists in the large group. Figure 1 shows the conceptual diagram for selecting Criteria 1 and 2. Here, GTV70, CTV70, PTV70, and the oral cavity, which were the closest to the dental metal artifact area in OARs, were evaluated in terms of mean dose. For PTV70, D98% and D2% — encompassing 98% and 2% of the PTV, respectively — were evaluated in addition to the mean dose to assess dose coverage. In addition, the oral cavity was evaluated along with the dose–volume histograms (DVHs) of physician F, the contour of Criterion 1, and physician D, the contour of Criterion 2. Furthermore, a three‐dimensional gamma analysis (GA) was performed. The mean doses D98% and D2% of each assessment in the small and large groups were calculated using 3DVH; the two groups were compared using a t‐test. GA was also evaluated using 3 DVH, and the difference in dose distribution between Criterion 1 (Physician F) and the other five structure sets was evaluated. Similarly, the difference in dose distribution between Criterion 2 (Physician D) and the other five structure sets was also evaluated by GA. The GA criteria used were 2 mm/2%.

In addition, the dice similarity coefficient (DSC) was used to quantify the contouring of the dental metal artifact area for the metal artifact contours of Physician D, corresponding to Criterion 2, and the five radiation oncologists.

### 2.D Effect of different dental metal artifact conversion volumes on dose distribution

#### 2.D.1 Contouring of dental metal artifact area

**Fig 2:**
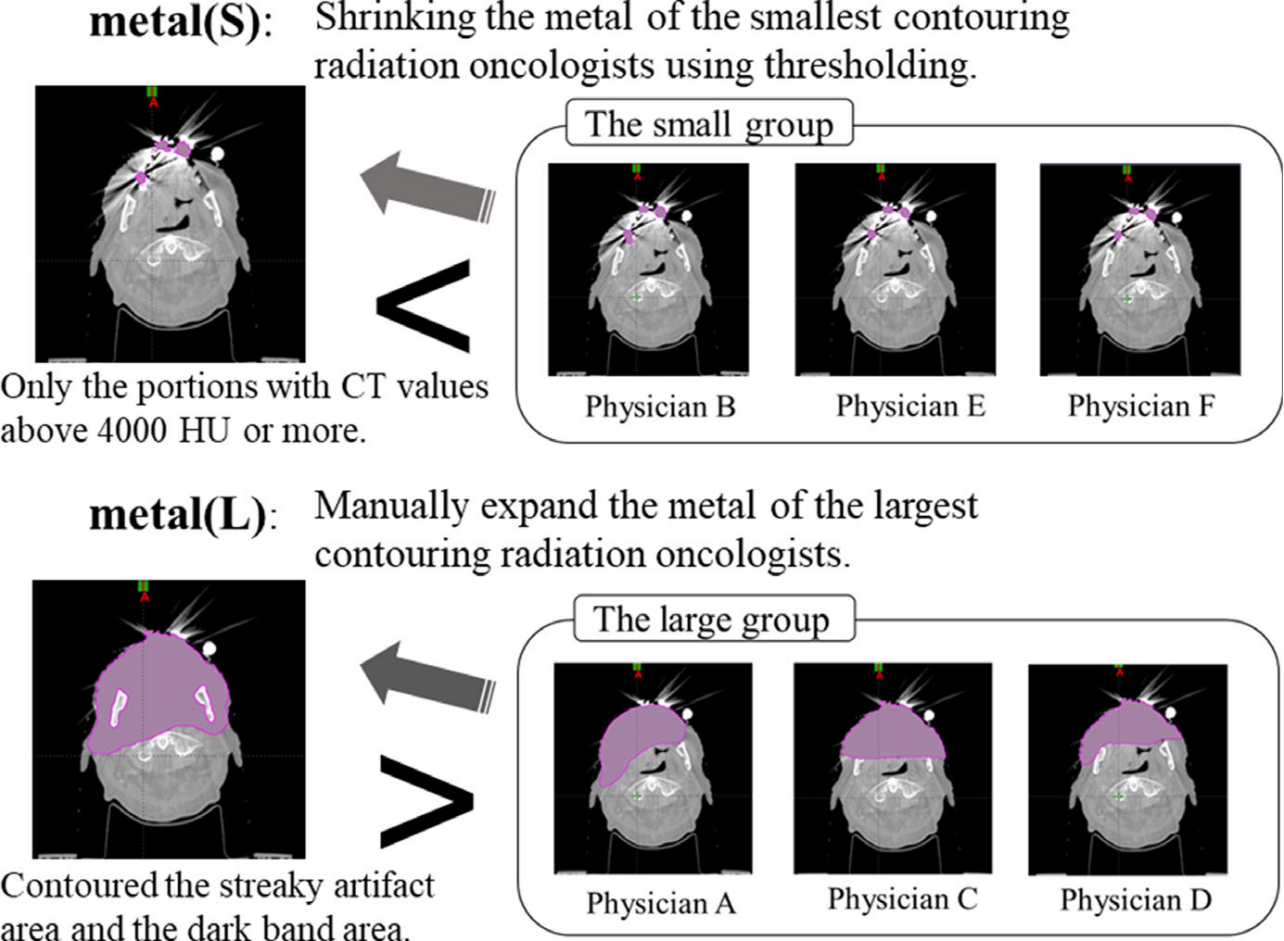
Creation concept of structure sets S and L

Based on the contouring determined by the method of Section 2.C, the contouring of the dental metal artifact area was also changed as an additional experiment. First, a set of the largest contouring areas of the structure metal (L) was created by expanding the metal of the radiation oncologist who had the largest contouring volume of the six radiation oncologists. The structure set L was created by manually contouring more than half of the anterior surface in the anterior–posterior direction, including the streak artifact area and the dark band area due to the denture. Here, because the mandible is a tissue that is not included in the denture artifact, it was excluded from the expanded structure metal (L). Next, a set of the smallest contouring areas of the structure metal (S) was created by shrinking the metal of the radiation oncologist who had the smallest contouring volume of the six radiation oncologists. With metal (S), only the portions with CT values above 4000 HU were automatically extracted at the threshold. A range shifter, which is a function of Eclipse, was used for threshold processing. The metal in (L) and (S) was replaced with CT values of water, and the other structures remained the original structures used in the actual radiotherapy (Fig. 2). In addition, a structure set N was created in which dental metal artifacts were not contoured.

#### 2.D.2 Recalculation of dose distribution

The dose distribution was transferred from the original treatment plan to the structure sets L, S, and N, and recalculation was performed. As in Section 2.C, the isocenters of the original treatment plan and the three structure sets were the same; only the dose distribution was transposed.

#### 2.D.3 Evaluation method

The dose distributions of the structure sets L and S and the dose distributions of structure sets L and N were evaluated using 3DVH with the three‐dimensional GA criteria of 2 mm/2%. The subjects of the evaluation were GTV70, CTV70, PTV70, and oral cavity.

In addition, the volume of PTV70, the overlap of PTV70 and metal in (L), and the overlap of PTV70 and metal in (S) were calculated on the TPS to carry out additional investigation in cases with a low GA pass rate. We divided the cases into groups with low GA pass rates and other cases, and compared the differences in volume using the Mann–Whitney U test.

## 3 Results

### 3.A Difference in dose distribution due to inter‐observer variability

Table 1 shows the mean dose and t‐test results for each evaluation subject in the small and large groups; only PTV70 was evaluated for D98% and D2%. The numerical values shown are the mean dose ± standard deviation. A significant difference (*P* < 0.001) was found in the mean doses of GTV70 and CTV70; the mean dose in the large group was higher than that in the small group. For PTV70, all endpoints were significantly different (*P* < 0.001), and the mean dose in the large group was higher than that in the small group. No significant difference was observed in the oral cavity (*P* = 0.78).

**Table 1:** Mean dose and standard deviation of the small group and the large group and test results.

Evaluation structure	The small group	The large group	*P*‐value
GTV70 (mean dose)	72.94 ± 0.71	73.13 ± 0.67	<0.001
CTV70 (mean dose)	73.13 ± 0.61	73.34 ± 0.59	<0.001
PTV70 (mean dose)	72.73 ± 0.49	72.91 ± 0.47	<0.001
PTV70 (D98%)	68.83 ± 0.33	69.06 ± 0.33	<0.001
PTV70 (D2%)	75.65 ± 0.78	75.81 ± 0.77	<0.001
Oral cavity (mean dose)	47.99 ± 12.61	47.89 ± 12.46	0.78

GTV: gross tumor volume, CTV: clinical target volume, PTV: planning target volume.

**Fig 3:**
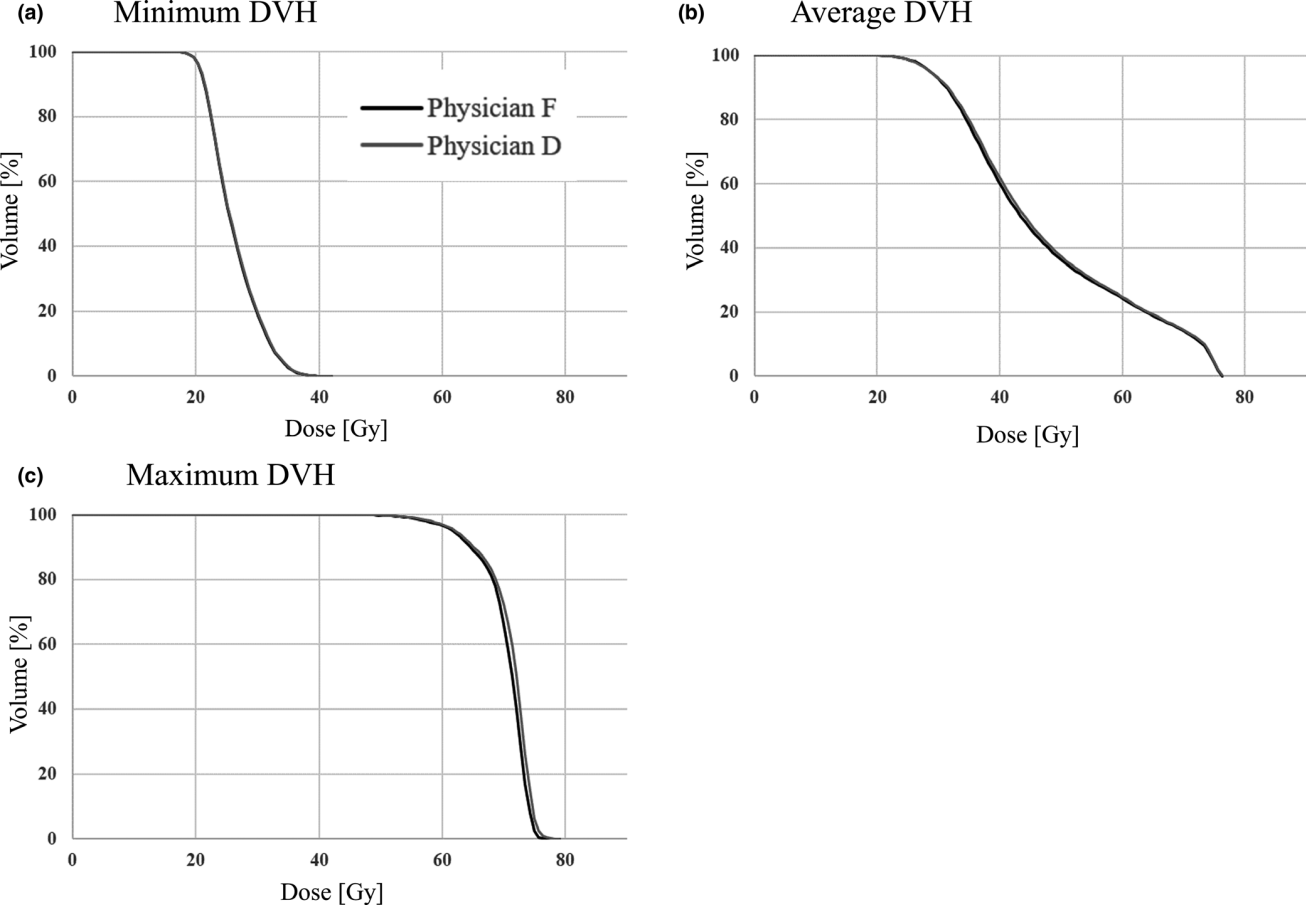
Minimum, average, and maximum dose volume histogram (DVH) differences in the oral cavities between the small and large groups. (a) Minimum DVH, (b) average DVH, (c) maximum DVH.

The minimum, average, and maximum DVH differences between the small and large oral cavity groups are shown in Fig. 3. The difference between the small and large groups tended to increase as the dose to the oral cavity increased.

Table 2 shows the results of GA for the dose distribution of Physician F, which is Criterion 1, and the dose distribution of the other five structure sets. The dose distribution for Criterion 1 differs little when compared to the dose distributions for Physicians B and E in the small group. Therefore, in Table 2, the GA pass rate of the small group was 100% in all evaluation subjects except PTV70 and oral cavity of Physician E. Physician E’s PTV70 and oral cavity GA pass rates were above 99.9% on average. The lowest pass rate among the large group Physicians A, C, and D in Criterion 1 was Physician A’s CTV70, with a mean pass rate of 97.6%. The oral cavity, which was considered closer to the dental metal artifact area than GTV70, CTV70, and PTV70, did not differ from the other evaluations.

**Table 2:** Criterion 1 and other physician dose distribution gamma analysis (GA) results. Results are shown as the average ± standard deviation of the pass rate.

Evaluation structure	Physician A	Physician B	Physician C	Physician D	Physician E
GTV70	98.00 ± 5.75	100.00 ± 0.00	98.46 ± 4.62	98.94 ± 3.05	100.00 ± 0.00
CTV70	97.63 ± 4.94	100.00 ± 0.00	98.40 ± 3.82	98.60 ± 2.99	100.00 ± 0.00
PTV70	97.88 ± 4.21	100.00 ± 0.00	98.53 ± 3.13	98.74 ± 2.49	99.94 ± 0.00
Oral cavity	98.06 ± 3.65	100.00 ± 0.00	98.25 ± 3.86	98.76 ± 2.47	99.92 ± 0.04

GTV: gross tumor volume, CTV: clinical target volume, PTV: planning target volume.

Table 3 shows the GA results of the dose distribution of Physician D, which is Criterion 2, and the dose distribution of the other five structure sets. The dose distribution for Criterion 2 differs little when compared to the dose distribution for Physicians A and C in the large group. Therefore, as shown in Table 3, the pass rate of the large group was 100% for all evaluation subjects except CTV70 and PTV70 of Physician C. Comparing the dose distribution of Criterion 2 with the dose distributions of Physicians B, E, and F in the small group, the lowest average GA pass rate was 98.3%, for CTV70 of Physician E.

**Table 3:** Criterion 2 and other physician dose distribution gamma analysis (GA) results. Results are shown as the average ± standard deviation of the pass rate.

Evaluation structure	Physician A	Physician B	Physician C	Physician E	Physician F
GTV70	100.00 ± 0.00	99.07 ± 2.59	100.00 ± 0.00	98.45 ± 4.47	98.77 ± 3.37
CTV70	100.00 ± 0.00	98.76 ± 2.72	99.99 ± 0.03	98.26 ± 3.76	98.51 ± 3.07
PTV70	100.00 ± 0.00	99.17 ± 1.75	99.99 ± 0.02	98.77 ± 2.41	98.98 ± 2.01
Oral cavity	100.00 ± 0.00	99.29 ± 1.53	100.00 ± 0.00	98.98 ± 2.20	99.04 ± 2.22

GTV: gross tumor volume, CTV: clinical target volume, PTV: planning target volume.

Table 4 lists the DSC results for Physician D and five radiation oncologists. In the case of Physician D, the criterion for the large group, the contour was similar to that obtained from Physician A, one of the other two physicians in the large group, for the metal artifacts; in this case, the mean DSC was 0.799. Furthermore, Physician C had the greatest variability in the DSC. Physicians B, E, and F in the small group tended to have less variability in the DSC than the radiation oncologists in the large group.

**Table 4:** Dice similarity coefficient (DSC) results for physician D and five radiation oncologists.

Case Number	Physician A vs Physician D	Physician B vs Physician D	Physician C vs Physician D	Physician E vs Physician D	Physician F vs Physician D
Case1	0.773	0.059	0.788	0.057	0.041
Case2	0.787	0.095	0.777	0.090	0.121
Case3	0.876	0.123	0.854	0.117	0.124
Case4	0.875	0.059	0.728	0.075	0.070
Case5	0.783	0.066	0.699	0.078	0.062
Case6	0.848	0.098	0.794	0.098	0.136
Case7	0.812	0.098	0.810	0.102	0.145
Case8	0.824	0.068	0.493	0.074	0.087
Case9	0.881	0.168	0.711	0.133	0.148
Case10	0.803	0.128	0.666	0.098	0.118
Case11	0.767	0.150	0.700	0.121	0.152
Case12	0.810	0.066	0.697	0.069	0.064
Case13	0.742	0.237	0.630	0.245	0.242
Case14	0.863	0.047	0.828	0.049	0.061
Case15	0.837	0.165	0.682	0.128	0.136
Case16	0.815	0.095	0.663	0.078	0.106
Case17	0.838	0.120	0.629	0.112	0.139
Case18	0.838	0.085	0.675	0.090	0.126
Case19	0.786	0.144	0.830	0.109	0.119
Case20	0.799	0.083	0.672	0.079	0.097
Case21	0.789	0.091	0.469	0.070	0.085
Case22	0.846	0.122	0.771	0.099	0.117
Case23	0.742	0.072	0.689	0.055	0.068
Case24	0.807	0.165	0.765	0.122	0.149
Case25	0.433	0.117	0.246	0.117	0.142
Mean	0.799	0.109	0.691	0.099	0.114
Standard deviation	0.085	0.045	0.132	0.039	0.042

### 3.B Effect of dental metal artifact conversion volume on dose distribution

**Fig 4:**
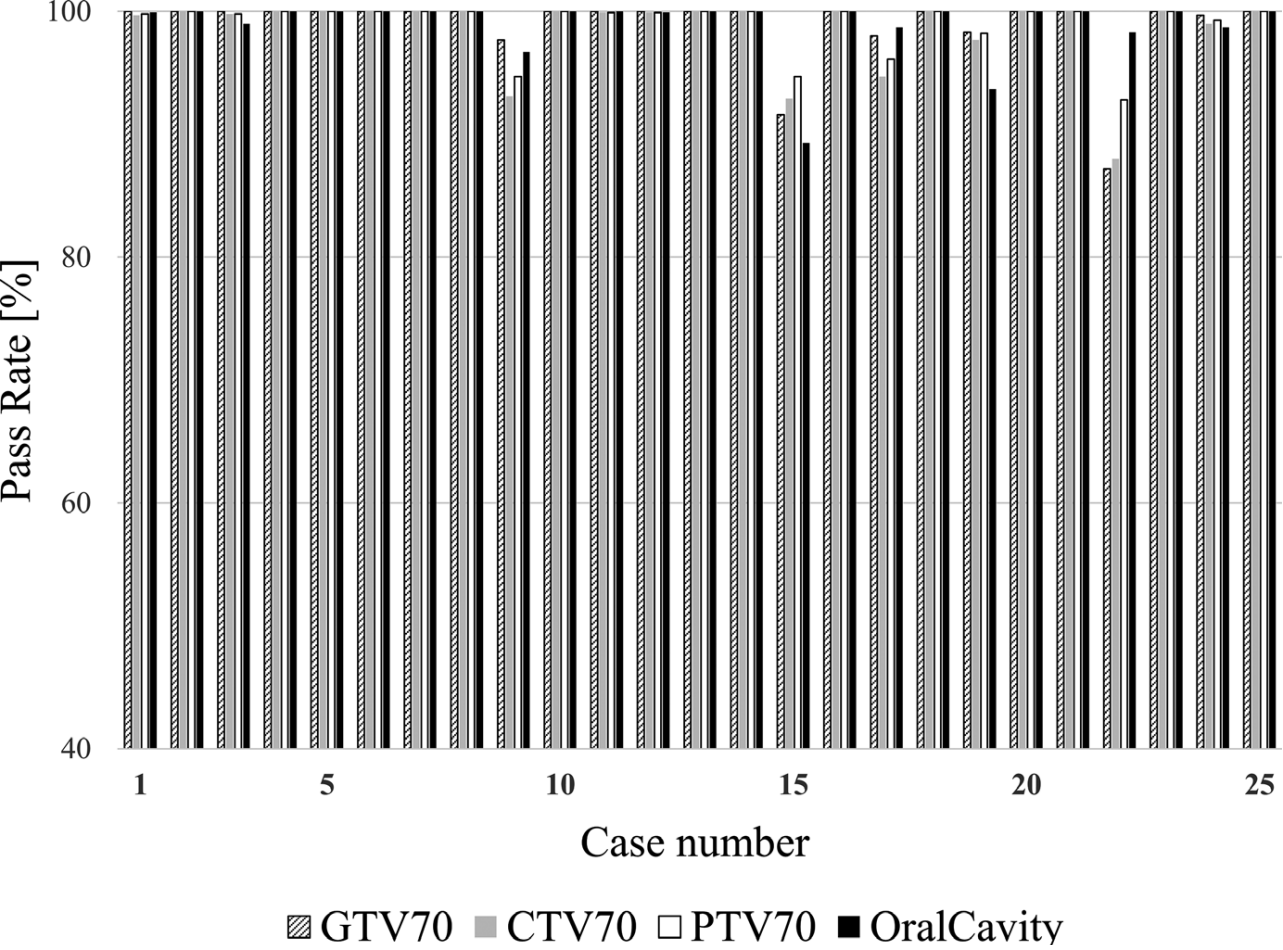
Results for gamma analysis for 2 mm/2% of the dose distribution obtained by recalculating using structure sets L and S, respectively.

Figure 4 shows the GA results of the difference between the dose plans transferred from the original treatment plan to the structure sets L and S and recalculated. Eighteen of the 25 cases had a 100% pass rate for all evaluations. In Case 22 GTV70 and CTV70, the GA pass rate was less than 90% (87.2% and 88.0%, respectively). The case with the lowest PTV70 pass rate was Case 22, for which the pass rate was 92.8%. The lowest oral cavity pass rate was found in Case 15, with a pass rate of 89.3%. The pass rate was 93% or higher in the other cases. The evaluation subject with the highest average pass rate was PTV70 at 99.0%, and the evaluation subject with the lowest average pass rate was CTV70 at 98.6%.

**Fig 5:**
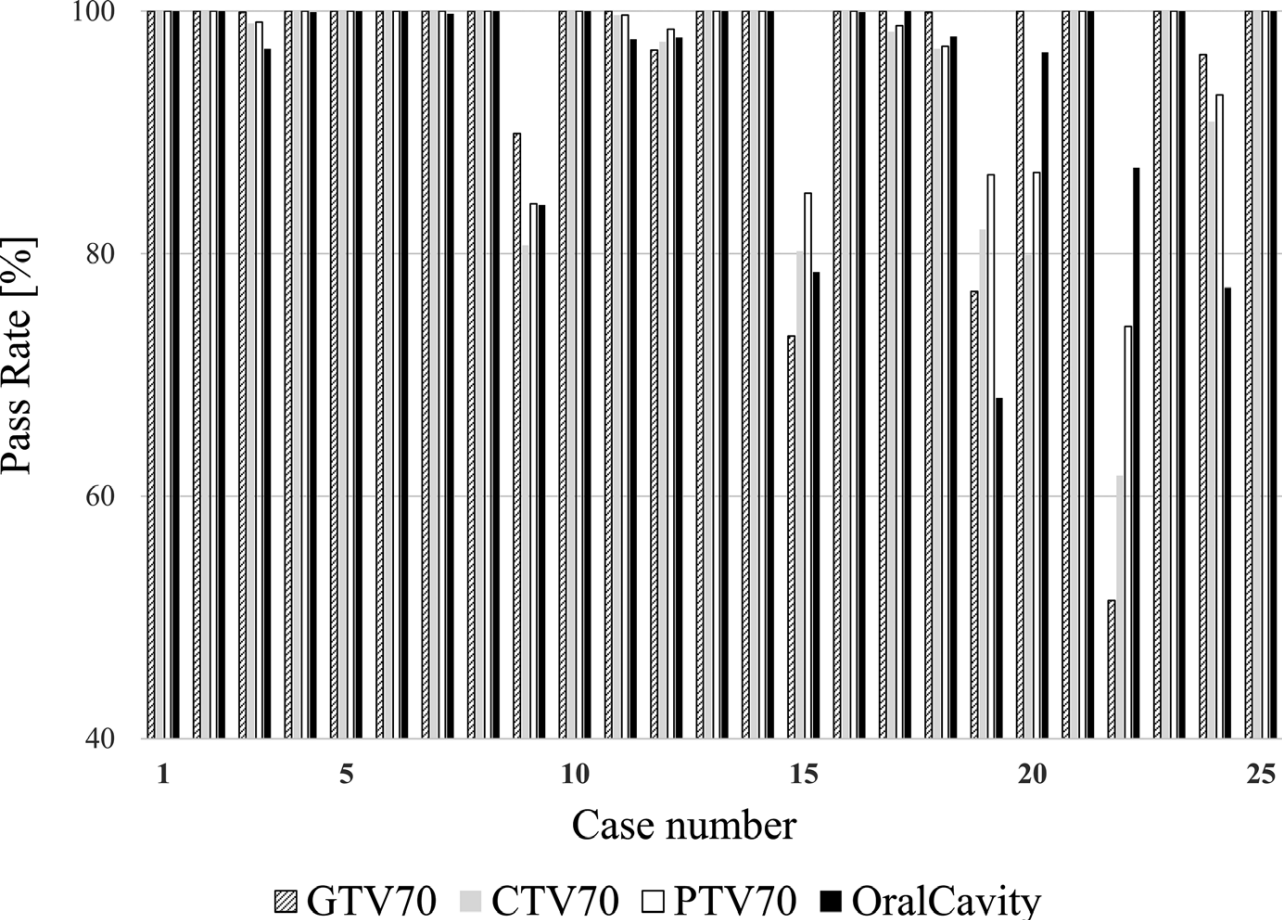
Results for gamma analysis for 2 mm/2% of the dose distribution obtained by recalculating using structure sets L and N, respectively.

Figure 5 shows the GA results of the difference between the dose plans transferred from the original treatment plan to the structure sets L and N and recalculated. In 14 of the 25 cases, the pass rate was 100% in all subjects. The pass rate was 90% or less in all the subjects of the evaluation of Case 9, Case 15, Case 19, and Case 22. In Case 22, the pass rates of GTV70, CTV70, PTV70, and oral cavity were 51.4%, 61.7%, 74.0%, and 87.1%, respectively, which were the lowest values of the cases studied. The subject of the evaluation with the highest average pass rate was PTV70 with 96.1%, and CTV70 was the lowest with 94.7%.

As an additional investigation, Table 5 shows the volumes of PTV70, PTV70, and metal (L) overlap, and PTV70 and metal (S) overlap in four cases with a GA pass rate of 90% or less. In Case 19 only, there was no overlap between PTV70 and metal S. The overlap volume of Case 19 PTV70 and metal (L) was similar to the other three cases. In Case 19, the volume of PTV70 was smaller than that in the other three cases. Table 6 shows the results of the significant difference test of the volume in each of the four cases with a GA pass rate of 90% or less, as shown in Table 5, and the 21 cases with a GA pass rate of 90% or more. There was a significant difference in the volume of overlap between PTV70 and metal (L) and the volume of overlap between PTV70 and metal (S).

**Table 5:** Overlap volume of PTV70 and structure metal in S and L and volume of PTV70 in four cases with low pass rates in gamma analysis (GA).

Case number	Overlap(PTV ∩ metal)[cc]		PTV70[cc]
	(S)	(L)	
9	0.3	24.3	130.7
15	1.7	26.5	271.1
19	0.0	24.5	49.2
22	0.7	28.1	109.9

PTV: planning target volume

**Table 6:** Results of volume comparison and tests for each evaluation target in four cases with low pass rates in gamma analysis (GA) and other cases. The results are shown as mean ± standard deviation.

Evaluation structure	Volume [cc]		*p*‐value
	Four cases with low pass rate	Other 21 cases	
overlap(S)*	1.20 ± 0.78	0.09 ± 0.38	0.0001^†^
overlap(L)	27.93 ± 3.17	7.52 ± 10.86	0.003^‡^
PTV70	194.78 ± 54.06	146.57 ± 84.15	0.18

* Overlap(S): overlap between PTV70 and metal(S).

^†^ <0.001.

^‡^ <0.05.

## 4 Discussion

Wang et al.^13^ suggested that the PTV dose may be reduced by the denture attenuation effect in head‐and‐neck IMRT. Mail et al.^14^ reported that in VMAT of the head‐and‐neck, the backscatter dose increases in the presence of dentures, and dose perturbation occurs, which affects the dose distribution. Kim et al.^7^ reported that denture artifacts could lead to hot spots in the OAR and cold spots in each target volume. Furthermore, Parenica et al.^15^ reported an increased dose in OARs affected by streak artifacts from dentures. Although they mention the physical dose distribution effect of dental metal artifacts, they do not report the effect of artificial variation of dental metal artifact contouring on the dose distribution during head‐and‐neck VMAT planning.

In this study, the mean doses of the small group that contoured only dentures and the large group that contoured streak artifacts and dark band regions for GTV70, CTV70, and PTV70 are shown in Table 1. There was a significant difference in the subjects of the evaluation. It was also found that the mean dose in the large group was slightly higher than that in the small group. The small group did not contour the areas where CT values are nonuniform, such as dark band areas, which do not actually exist in the body. It is considered that a slight difference in the mean dose was caused by the difference in dose calculation due to the existence of this nonuniform region. This is consistent with the reports by Wang et al.^13^ and Kim et al.^7^, and it is considered that there was a slight dose reduction in the target volume due to denture artifacts. No difference in the dose distribution between the large and small groups was found in the dose distribution GA evaluation. In addition, the comparison of the dose distributions between groups showed that the average pass rate was 97% or more, indicating a high agreement in the dose distributions in all the evaluation subjects. This suggests that the artificial distribution of dental metal artifact contouring has little effect on the dose distribution.

Maerz et al.^16^ reported that VMAT has a higher dose calculation accuracy than IMRT when a denture is present. In the case of IMRT, multiple segments are irradiated from a single direction. Therefore, the beam segment that intersects the denture is irradiated in the same direction through the denture. In the case of VMAT, however, because the beam is continuously emitted from various directions, the dose perturbation caused by the beam passing through the denture has little effect on the dose distribution. From this, it is considered that the artificial distribution of artificial tooth contouring has little effect on dose distribution.

As a result of comparing the dose distributions recalculated from the structure sets L and S created by changing the volume of dental metal artifact contouring by GA, there were few cases in which the pass rate significantly decreased, and the average pass rate was 98.5% or more for all evaluation items. In addition, among the evaluation items, there was no difference between the tendency of the GA pass rate of the oral cavity, which was closest to the dental metal artifact region, and that of the other evaluation items.

The DSC was used to assess geometric differences between contours within each group quantitatively. From the American Association of Physicists in Medicine Task Group132 guidelines,^17^ a contour agreement of 0.8 or better obtained using the DSC is considered a good match. A previous study reported^18^ the difference between feature points and DSCs using treatment‐planning CT and cone‐beam CT obtained immediately before the treatment to assess the concordance rate of GTV contours of breath‐holding in lung cancer patients. A DSC of ~ 0.7 has been reported to indicate a feature point shift of 2–3 mm in three dimensions. It is understandable that the DSC of ~ 0.7 in this study is more than 99% with a GA criterion of 2 mm/2%. In addition, the DSC results of Physicians B, E, and F in the small group exhibited less case‐to‐case variation than those of the physicians in the large group. This can be attributed to the fact that the small group contoured only metal artifacts, and therefore, the contouring was less ambiguous than that in the large group, which included the dark band. The contour of metal artifacts for the large group — Physicians A and D — was comparable based on the DSC results; however, Physicians C and D had DSCs below 0.5 in some cases. The difference in the dose distribution between the two contours was more than 99% for all contours based on the GA results, thereby suggesting that the effect on the dose distribution was minimal. This finding suggests that the influence of PTV size and geometric arrangement on the dose distribution is greater than the anthropogenic influence on the contouring of metal artifacts. The magnitude of the effect of PTV size and geometric arrangement on dose distribution can be understood from the DVH of the oral cavity shown in Figure 3, where the dose difference between the small and large groups tends to increase as the oral dose increases.

In contrast, from the results in Figure 4, in the dose distribution comparison between the structure set L and structure set N in which the dental metal artifact region was not contoured, there were cases in which the GA pass rate significantly decreased. CTV70 had the lowest average pass rate for evaluation at 94.7%. The lowest GA pass rate was 51.4% for Case 22 GTV70, which was a large decrease. Cases with relatively low GA pass rates in the structure sets L and S shown in Figure 3 tended to have lower pass rates in L and N, as shown in Figure 4. Therefore, performing contouring of dental metal artifacts and substituting the area with CT values of water is a necessary task to implement the VMAT treatment plan.

The structure set L was based on the CT for treatment planning and was manually contoured to every corner including the streak artifact area and the dark band area due to the denture. In contrast, in the structure set S, the location of the denture was set as the region of interest, and only the area above 4000 HU was contoured with threshold processing using the range shifter feature of Eclipse. The structure set S can be created more easily than L. Therefore, contouring of dental metal artifacts by thresholding may be useful in actual medical care.

From Table 6, the pass ratio tends to decrease as the overlap volume of PTV70 and the dental metal artifact region increases. In other words, it is conceivable that the distance and the positional relationship between the metal artifact of the denture and the respective evaluation subjects are related to the pass rate. This can be explained by the dose perturbation on the upstream side and the downstream side of x‐ray incidence on the denture reported by Mail et al.^14^ Because stronger dose perturbations occur near the denture surface, the number and extent of dentures as well as the positions of the denture and each assessment target due to the angle of the jaw during treatment planning CT scans affect the GA pass rate.

Recently, an increasing number of reports have carried out dose calculation in VMAT using an image reconstruction algorithm that enables the reduction of metal artifacts in the image reconstruction stage of CT.^19^, ^20^, ^21^ Hansen et al.^21^ reported that the consistency of contouring was improved by planning treatment using CT images using MAR. However, there was no significant difference in the dose distribution of CTV and PTV.^21^ The current study investigated the effect of anthropogenic variation in dental metal artifact contouring on dose distribution in a facility with an MAR‐free environment. This is important information for planning head‐and‐neck VMAT treatment for facilities that cannot use MAR. In the current study, we used AAA based on the dose kernel in water as a dose calculation algorithm. The dose calculation algorithms implemented in the TPS used in this study include Acuros XB10 (AXB10) and Acuros XB11 (AXB11) in addition to AAA. Previous reports have stated that AAA causes dose errors in nonuniform areas.^22^ In the future, we will investigate the effect of the difference in the volume of mass conversion of dental metal artifacts on the dose distribution by switching to AXB10, which has a smaller dose error in nonuniform regions, and AXB11,^23^ which has a shorter computation time in VMAT dose calculation.

## 5 Conclusions

Our results show that the effect of artificial variation in manual dental metal artifact contouring on dose distribution is small. However, it is important to note that the dose distribution may change depending on the contouring method in cases where the overlap between PTV and the metal artifacts is large. Based on the small variation seen in the dose distribution, the method of thresholding the portion of the denture with a CT value of 4000 HU or higher is simpler and more efficient than manually contouring dental metal artifacts.

## Conflict of Interest

No conflicts of interest.
